# A discrete Single Delay Model for the Intra-Venous Glucose Tolerance Test

**DOI:** 10.1186/1742-4682-4-35

**Published:** 2007-09-12

**Authors:** Simona Panunzi, Pasquale Palumbo, Andrea De Gaetano

**Affiliations:** 1 CNR-IASI BioMatLab, Largo A. Gemelli 8 – 00168 Rome, Italy.; 2 CNR-IASI, Viale A. Manzoni 30 – 00185 Rome, Italy.

## Abstract

**Background:**

Due to the increasing importance of identifying insulin resistance, a need exists to have a reliable mathematical model representing the glucose/insulin control system. Such a model should be simple enough to allow precise estimation of insulin sensitivity on a single patient, yet exhibit stable dynamics and reproduce accepted physiological behavior.

**Results:**

A new, discrete Single Delay Model (SDM) of the glucose/insulin system is proposed, applicable to Intra-Venous Glucose Tolerance Tests (IVGTTs) as well as to multiple injection and infusion schemes, which is fitted to both glucose and insulin observations simultaneously. The SDM is stable around baseline equilibrium values and has positive bounded solutions at all times. Applying a similar definition as for the Minimal Model (MM) S_I _index, insulin sensitivity is directly represented by the free parameter K_xgI _of the SDM.

In order to assess the reliability of Insulin Sensitivity determinations, both SDM and MM have been fitted to 40 IVGTTs from healthy volunteers. Precision of all parameter estimates is better with the SDM: 40 out of 40 subjects showed identifiable (CV < 52%) K_xgI _from the SDM, 20 out of 40 having identifiable S_I _from the MM. K_xgI _correlates well with the inverse of the HOMA-IR index, while S_I _correlates only when excluding five subjects with extreme S_I _values. With the exception of these five subjects, the SDM and MM derived indices correlate very well (r = 0.93).

**Conclusion:**

The SDM is theoretically sound and practically robust, and can routinely be considered for the determination of insulin sensitivity from the IVGTT. Free software for estimating the SDM parameters is available.

## Background

The measurement of insulin sensitivity in humans from a relatively non-invasive test procedure is being felt as a pressing need, heightened in particular by the increase in the social cost of obesity-related dysmetabolic diseases [1-8]. Two experimental procedures are in general use for the estimation of insulin sensitivity: the Intra-Venous Glucose Tolerance Test (IVGTT), often modeled by means of the so-called Minimal Model (MM) [9,10], and the Euglycemic Hyperinsulinemic Clamp (EHC) [11]. The EHC is often considered the "*gold standard*" for the determination of insulin resistance. However, the standard IVGTT is simpler to perform, carries no significant associated risk and delivers potentially richer information content. The difficulty with using the IVGTT is its interpretation, for which it is necessary to apply a mathematical model of the status of the negative feedback regulation of glucose and insulin on each other in the studied experimental subject.

Due to its relatively simple structure and to its great clinical importance, the glucose/insulin system has been the object of repeated mathematical modeling attempts [12-30]. The mere fact that several models have been proposed shows that mathematical, statistical and physiological considerations have to be carefully integrated when attempting to represent the glucose/insulin system. In modeling the IVGTT, we require a reasonably simple model, with as few parameters to be estimated as practicable, and with a qualitative behavior consistent with physiology. Further, the model formulation, while applicable to the standard IVGTT, should logically and easily extend to model other often envisaged experimental procedures, like repeated glucose boli, or infusions. A simple, discrete Single Delay Model ("the discrete SDM") of both feedback control arms of the glucose-insulin system during an IVGTT has already been validated as far as its formal properties are concerned [31,32].

The present work has three main goals. The first goal is to present the physiological assumptions underlying the new model, from which an insulin sensitivity index, consistent with the currently employed insulin sensitivity index from the Minimal Model, can be derived. The second goal is to discuss in general the inconsistent results obtained by means of the common procedure of using observed insulinemias for the estimation of the glucose kinetics and then using observed glycemias for the estimation of insulin kinetics (instead of performing a single optimization on both feedback control arms of the glucose/insulin system). The third goal is finally to study comparatively the indices of Insulin sensitivity which are obtained from the newly proposed SDM and from the Minimal Model in its standard formulation (two equations for glycemia, driven by interpolated observed insulinemias), on a sample of IVGTT's from 40 healthy volunteers.

## Methods

### Experimental protocol

Data from 40 healthy volunteers (18 males and 22 females, average anthropometric characteristics reported in Table 1), who had been previously studied in several protocols at the Catholic University Department of Metabolic Diseases were analyzed. All subjects had negative family and personal histories for Diabetes Mellitus and other endocrine diseases, were on no medications, had no current illness and had maintained a constant body weight for the six months preceding each study. For the three days preceding the study each subject followed a standard composition diet (55% carbohydrate, 30% fat, 15% protein) ad libitum with at least 250 g carbohydrates per day. Written informed consent was obtained in all cases; all original study protocols were conducted according to the Declaration of Helsinki and along the guidelines of the institutional review board of the Catholic University School of Medicine, Rome, Italy.

**Table 1:** Anthropometric characteristics of the subjects studied (mean ± SD in 40 subjects).

**Gb (mM)**	**Ib (pM)**	**Gender n (%)**	**Age (years)**	**Height (cm)**	**BW (kg)**	**BMI (Kg/m^2^)**
		F 22 (55%)				
4.54 ± 0.51	40.80 ± 21.88	M 18 (45%)	45.25 ± 16.44	166.10 ± 8.63	67.53 ± 10.01	24.36 ± 2.34

Each study was performed at 8:00 AM, after an overnight fast, with the subject supine in a quiet room with constant temperature of 22–24°C. Bilateral polyethylene IV cannulas were inserted into antecubital veins. The standard IVGTT was employed (without either Tolbutamide or insulin injections)[9]: at time 0 (0') a 33% glucose solution (0.33 g Glucose/kg Body Weight) was rapidly injected (less than 3 minutes) through one arm line. Blood samples (3 ml each, in lithium heparin) were obtained at -30', -15', 0', 2', 4', 6', 8', 10', 12', 15', 20', 25', 30', 35', 40', 50', 60', 80', 100', 120', 140', 160' and 180' through the contralateral arm vein. Each sample was immediately centrifuged and plasma was separated. Plasma glucose was measured by the glucose oxidase method (Beckman Glucose Analyzer II, Beckman Instruments, Fullerton, CA, USA). Plasma insulin was assayed by standard radio immunoassay technique. The plasma levels of glucose and insulin obtained at -30', -15' and 0' were averaged to yield the baseline values referred to 0'.

### The discrete Single Delay Model

In the development of the discrete SDM, four two-compartment models, describing the variation in time of plasma glucose and plasma insulin concentrations following an IVGTT, have been considered.

For each model the glucose equation includes a second-order linear term describing insulin-dependent glucose uptake, expressed in net terms since it includes changes in liver glucose delivery and changes in glucose uptake, as well as a zero-order term expressing the net balance between a possible constant, insulin-independent fraction of hepatic glucose output and the essentially constant glucose utilization of the brain. A linear term for glucose tissue uptake may or may not be present, and the effect of plasma insulin on glucose kinetics may or may not be delayed.

Variations in plasma insulin concentration depend on the spontaneous decay of insulin and on pancreatic insulin secretion. After the nearly instantaneous first phase insulin secretion, represented in the model by means of the initial condition, a delay term is considered; it represents the pancreatic second phase secretion and formalizes the delay with which the pancreas responds to variations of glucose plasma concentrations.

**Figure 1:**
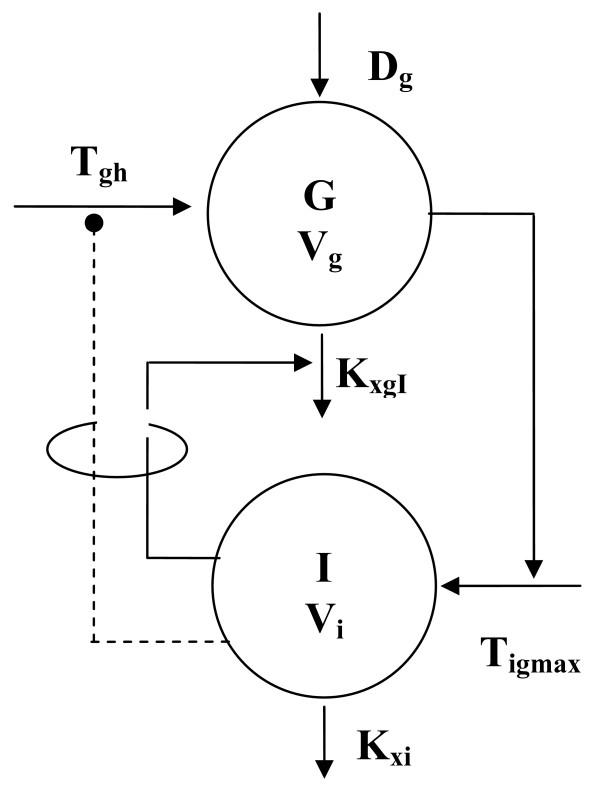
**Schematic representation of the two-compartments, one-discrete-delay model**. V_g _and V_i _are the distribution volumes respectively for Glucose (G) and Insulin (I). D_g _stands for the glucose bolus administered; K_xgI _is the second-order net elimination rate of glucose  per unit of insulin concentration; K_xi _is the first order elimination rate of insulin; T_gh _is the net difference between glucose production and glucose elimination; T_igmax _is the maximal rate of second phase insulin release.

The details of the four considered models are reported in Table 2. Each model was fitted to the experimentally observed concentrations and for each of the 40 subjects the Akaike value was computed. Models were compared by performing paired t-tests on the computed Akaike scores. The selected model was model A, whose schematic diagram is represented in Figure 1 and whose equations are reported below:

**Table 2:** Tested models and relative average Akaike information Criterion (AIC).

**Model**	**Desciption**	**Free parameters**	**Average AIC**
A	Without first order plasma glucose elimination (K_xg_) and without delay on insulin action (τ_i_)	V_g_, I_Δ_, τ_g_, K_xgI_, K_xi_, γ	383.90
B	With first order plasma glucose elimination (K_xg_) and without delay on insulin action (τ_i_)	V_g_, I_Δ_, τ_g_, K_xgI_, K_xi_, γ, K_xg_	386.72
C	Without first order plasma glucose elimination (Kxg) and with delay on insulin action (τ_i_)	V_g_, I_Δ_, τ_g_, K_xgI_, K_xi_, γ, K_xg_, τ_i_	385.95
D	With first order plasma glucose elimination (K_xg_) and with delay on insulin action (τ_i_)	V_g_, I_Δ_, τ_g_, K_xgI_, K_xi_, γ, K_xg_, K_xg_, τ_i_	389.03

The four models studied differ according to the presence or absence of an insulin-independent glucose elimination rate term (-K_xg _G) and according to the presence or absence of an explicit delay in the action of insulin in stimulating tissue glucose uptake (I(t-τ_i_) instead of I(t)). The model that does not include either one of these two features was named model A; model B includes the term (-K_xg_G); model C uses I(t-τ_i_) instead of I(t); model D includes both.

dG(t)dt=−KxgII(t)G(t)+TghVg
 MathType@MTEF@5@5@+=feaafiart1ev1aaatCvAUfKttLearuWrP9MDH5MBPbIqV92AaeXatLxBI9gBaebbnrfifHhDYfgasaacH8akY=wiFfYdH8Gipec8Eeeu0xXdbba9frFj0=OqFfea0dXdd9vqai=hGuQ8kuc9pgc9s8qqaq=dirpe0xb9q8qiLsFr0=vr0=vr0dc8meaabaqaciaacaGaaeqabaqabeGadaaakeaadaWcaaqaaiabbsgaKjabbEeahnaabmaabaGaeeiDaqhacaGLOaGaayzkaaaabaGaeeizaqMaeeiDaqhaaiabg2da9iabgkHiTiabbUealnaaBaaaleaacqqG4baEcqqGNbWzcqqGjbqsaeqaaOGaeeysaK0aaeWaaeaacqqG0baDaiaawIcacaGLPaaacqqGhbWrdaqadaqaaiabbsha0bGaayjkaiaawMcaaiabgUcaRmaalaaabaGaeeivaq1aaSbaaSqaaiabbEgaNjabbIgaObqabaaakeaacqqGwbGvdaWgaaWcbaGaee4zaCgabeaaaaaaaa@4BD6@

G(t)≡Gb∀t∈(−∞,0),G(0)=Gb+GΔ,where GΔ=DgVg
 MathType@MTEF@5@5@+=feaafiart1ev1aaatCvAUfKttLearuWrP9MDH5MBPbIqV92AaeXatLxBI9gBaebbnrfifHhDYfgasaacH8akY=wiFfYdH8Gipec8Eeeu0xXdbba9frFj0=OqFfea0dXdd9vqai=hGuQ8kuc9pgc9s8qqaq=dirpe0xb9q8qiLsFr0=vr0=vr0dc8meaabaqaciaacaGaaeqabaqabeGadaaakeaafaqabeqadaaabaGaee4raC0aaeWaaeaacqqG0baDaiaawIcacaGLPaaacqGHHjIUcqqGhbWrdaWgaaWcbaGaeeOyaigabeaakiabgcGiIiabbsha0jabgIGiopaabmaabaGaeyOeI0IaeyOhIuQaeiilaWIaeGimaadacaGLOaGaayzkaaGaeiilaWcabaGaee4raC0aaeWaaeaacqaIWaamaiaawIcacaGLPaaacqGH9aqpcqqGhbWrdaWgaaWcbaGaeeOyaigabeaakiabgUcaRiabbEeahnaaBaaaleaacqqHuoaraeqaaOGaeiilaWcabaGaee4DaCNaeeiAaGMaeeyzauMaeeOCaiNaeeyzauMaeeiiaaIaee4raC0aaSbaaSqaaiabfs5aebqabaGccqGH9aqpdaWcaaqaaiabbseaenaaBaaaleaacqqGNbWzaeqaaaGcbaGaeeOvay1aaSbaaSqaaiabbEgaNbqabaaaaaaaaaa@5BCF@

dI(t)dt=−KxiI(t)+Tigmax⁡Vi(G(t−τg)G∗)γ1+(G(t−τg)G∗)γ
 MathType@MTEF@5@5@+=feaafiart1ev1aaatCvAUfKttLearuWrP9MDH5MBPbIqV92AaeXatLxBI9gBaebbnrfifHhDYfgasaacH8akY=wiFfYdH8Gipec8Eeeu0xXdbba9frFj0=OqFfea0dXdd9vqai=hGuQ8kuc9pgc9s8qqaq=dirpe0xb9q8qiLsFr0=vr0=vr0dc8meaabaqaciaacaGaaeqabaqabeGadaaakeaadaWcaaqaaiabbsgaKjabbMeajnaabmaabaGaeeiDaqhacaGLOaGaayzkaaaabaGaeeizaqMaeeiDaqhaaiabg2da9iabgkHiTiabbUealnaaBaaaleaacqqG4baEcqqGPbqAaeqaaOGaeeysaK0aaeWaaeaacqqG0baDaiaawIcacaGLPaaacqGHRaWkdaWcaaqaaiabbsfaunaaBaaaleaacqqGPbqAcqqGNbWzcyGGTbqBcqGGHbqycqGG4baEaeqaaaGcbaGaeeOvay1aaSbaaSqaaiabbMgaPbqabaaaaOWaaSaaaeaadaqadaqaamaalaaabaGaee4raC0aaeWaaeaacqqG0baDcqGHsislcqaHepaDdaWgaaWcbaGaee4zaCgabeaaaOGaayjkaiaawMcaaaqaaiabbEeahnaaCaaaleqabaGaey4fIOcaaaaaaOGaayjkaiaawMcaamaaCaaaleqabaGaeq4SdCgaaaGcbaGaeGymaeJaey4kaSYaaeWaaeaadaWcaaqaaiabbEeahnaabmaabaGaeeiDaqNaeyOeI0IaeqiXdq3aaSbaaSqaaiabbEgaNbqabaaakiaawIcacaGLPaaaaeaacqqGhbWrdaahaaWcbeqaaiabgEHiQaaaaaaakiaawIcacaGLPaaadaahaaWcbeqaaiabeo7aNbaaaaaaaa@68BE@

I (0) = I_b _+ I_ΔG_G_Δ_,

The symbols are defined in Table 3. In equation (1) the term -K_xgI _(t) G (t) represents the net balance between insulin-dependent glucose uptake from peripheral tissues and insulin-dependent hepatic glucose output (above zero-order, constant hepatic glucose output), whereas the term TghVg
 MathType@MTEF@5@5@+=feaafiart1ev1aaatCvAUfKttLearuWrP9MDH5MBPbIqV92AaeXatLxBI9gBaebbnrfifHhDYfgasaacH8akY=wiFfYdH8Gipec8Eeeu0xXdbba9frFj0=OqFfea0dXdd9vqai=hGuQ8kuc9pgc9s8qqaq=dirpe0xb9q8qiLsFr0=vr0=vr0dc8meaabaqaciaacaGaaeqabaqabeGadaaakeaadaWcaaqaaiabbsfaunaaBaaaleaacqqGNbWzcqqGObaAaeqaaaGcbaGaeeOvay1aaSbaaSqaaiabbEgaNbqabaaaaaaa@3381@ represents the net difference between insulin-independent tissue glucose uptake (essentially from the brain) and the constant part of hepatic glucose output. The initial condition G_b _+ G_Δ _expresses the glucose concentration as the variation with respect to the basal condition, as a consequence of the IV glucose bolus. In the second equation, the first linear term -K_xi_I (t) represents spontaneous insulin degradation, whereas the second term represents second-phase insulin delivery from the β-cells. Its functional form is consistent with the hypothesis that insulin production is limited, reaching a maximal rate of release T_igmax_/V_i _by way of a Michaelis-Menten dynamics or a sigmoidal shape according to whether the γ value is 1 or greater than 1 respectively. Situations where γ is equal to zero correspond to a lack of response of the pancreas to variations of circulating glucose, while for γ values between zero and 1 the shape of the response resembles a Michaelis-Menten, with a sharper curvature towards the asymptote. The parameter γ expresses therefore the capability of the pancreas to accelerate its insulin secretion in response to progressively increasing blood glucose concentrations. The initial condition I_b _+ I_ΔG _G_Δ _represents instead the immediate first-phase response of the pancreas to the sudden increment in glucose plasma concentration.

**Table 3:** Definition of the symbols in the discrete Single Delay Model

**Symbol**	**Units**	**Definition**
t	[min]	time
G(t)	[mM]	glucose plasma concentration at time t
G_b_	[mM]	basal (preinjection) plasma glucose concentration
I(t)	[pM]	insulin plasma concentration at time t
I_b_	[pM]	basal (preinjection) insulin plasma concentration
K_xgI_	[min^-1 ^pM^-1^]	net rate of (insulin-dependent) glucose uptake by tissues per pM of plasma insulin concentration
T_gh_	[mmol min^-1 ^kgBW^-1^]	net balance of the constant fraction of hepatic glucose output (HGO) and insulin-independent zero-order glucose tissue uptake
V_g_	[L kgBW^-1^]	apparent distribution volume for glucose
D_g_	[mmol kgBW^-1^]	administered intravenous dose of glucose at time 0
G_Δ_	[mM]	theoretical increase in plasma glucose concentration over basal glucose concentration at time zero, after the instantaneous administration and distribution of the I.V. glucose bolus
K_xi_	[min^-1^]	apparent first-order disappearance rate constant for insulin
T_igmax_	[pmol min^-1^kgBW^-1^]	maximal rate of second-phase insulin release; at a glycemia equal to G* there corresponds an insulin secretion equal to T_igmax_/2
V_i_	[L kgBW^-1^]	apparent distribution volume for insulin
τ_g_	[min]	apparent delay with which the pancreas changes secondary insulin release in response to varying plasma glucose concentrations
γ	[#]	progressivity with which the pancreas reacts to circulating glucose concentrations. If γ were zero, the pancreas would not react to circulating glucose; if γ were 1, the pancreas would respond according to a Michaelis-Menten dynamics, with G* mM as the glucose concentration of half-maximal insulin secretion; if γ were greater than 1, the pancreas would respond according to a sigmoidal function, more and more sharply increasing as γ grows larger and larger
I_ΔG_	[pM mM^-1^]	first-phase insulin concentration increase per mM increase in glucose concentration at time zero due to the injected bolus
G*	[mM]	glycemia at which the insulin secretion rate is half of its maximum

It should be noticed that the form of Equation 1 is by no means new, a similar equation having been discussed, for instance in [33]. On the other hand, as far as we know, the form of Equation 2 is original. In particular, the exponent γ has been introduced to represent the 'acceleration' of pancreatic response with increasing glycemia, and has proved to be necessary for satisfactory model fit during model development.

From the steady state condition at baseline it follows that:

Tgh=KxgIIbGbVgandTigmax⁡=KxiIbVi[1+(GbG∗)γ]/(GbG∗)γ
 MathType@MTEF@5@5@+=feaafiart1ev1aaatCvAUfKttLearuWrP9MDH5MBPbIqV92AaeXatLxBI9gBaebbnrfifHhDYfgasaacH8akY=wiFfYdH8Gipec8Eeeu0xXdbba9frFj0=OqFfea0dXdd9vqai=hGuQ8kuc9pgc9s8qqaq=dirpe0xb9q8qiLsFr0=vr0=vr0dc8meaabaqaciaacaGaaeqabaqabeGadaaakeaafaqabeqadaaabaGaeeivaq1aaSbaaSqaaiabbEgaNjabbIgaObqabaGccqGH9aqpcqqGlbWsdaWgaaWcbaGaeeiEaGNaee4zaCMaeeysaKeabeaakiabbMeajnaaBaaaleaacqqGIbGyaeqaaOGaee4raC0aaSbaaSqaaiabbkgaIbqabaGccqqGwbGvdaWgaaWcbaGaee4zaCgabeaaaOqaaiabbggaHjabb6gaUjabbsgaKbqaaiabbsfaunaaBaaaleaacqqGPbqAcqqGNbWzcyGGTbqBcqGGHbqycqGG4baEaeqaaOGaeyypa0Jaee4saS0aaSbaaSqaaiabbIha4jabbMgaPbqabaGccqqGjbqsdaWgaaWcbaGaeeOyaigabeaakiabbAfawnaaBaaaleaacqqGPbqAaeqaaOWaaSGbaeaadaWadaqaaiabigdaXiabgUcaRmaabmaabaWaaSaaaeaacqqGhbWrdaWgaaWcbaGaeeOyaigabeaaaOqaaiabbEeahnaaCaaaleqabaGaey4fIOcaaaaaaOGaayjkaiaawMcaamaaCaaaleqabaGaeq4SdCgaaaGccaGLBbGaayzxaaaabaWaaeWaaeaadaWcaaqaaiabbEeahnaaBaaaleaacqqGIbGyaeqaaaGcbaGaee4raC0aaWbaaSqabeaacqGHxiIkaaaaaaGccaGLOaGaayzkaaWaaWbaaSqabeaacqaHZoWzaaaaaaaaaaa@6A11@

An index of insulin sensitivity may be easily derived from this model by applying the same definition as for the Minimal Model [9], i.e.

∂∂I [−∂∂G(dGdt)]=∂∂I[−∂∂G(−KxgIG(t)I(t)+TghVg)] =KxgI
 MathType@MTEF@5@5@+=feaafiart1ev1aaatCvAUfKttLearuWrP9MDH5MBPbIqV92AaeXatLxBI9gBaebbnrfifHhDYfgasaacH8akY=wiFfYdH8Gipec8Eeeu0xXdbba9frFj0=OqFfea0dXdd9vqai=hGuQ8kuc9pgc9s8qqaq=dirpe0xb9q8qiLsFr0=vr0=vr0dc8meaabaqaciaacaGaaeqabaqabeGadaaakeaadaWcaaqaaiabgkGi2cqaaiabgkGi2kabbMeajbaacqqGGaaidaWadaqaaiabgkHiTmaalaaabaGaeyOaIylabaGaeyOaIyRaee4raCeaamaabmaabaWaaSaaaeaacqqGKbazcqqGhbWraeaacqqGKbazcqqG0baDaaaacaGLOaGaayzkaaaacaGLBbGaayzxaaGaeyypa0ZaaSaaaeaacqGHciITaeaacqGHciITcqqGjbqsaaWaamWaaeaacqGHsisldaWcaaqaaiabgkGi2cqaaiabgkGi2kabbEeahbaadaqadaqaaiabgkHiTiabbUealnaaBaaaleaacqqG4baEcqqGNbWzcqqGjbqsaeqaaOGaee4raCKaeiikaGIaeeiDaqNaeiykaKIaeeysaKKaeiikaGIaeeiDaqNaeiykaKIaey4kaSYaaSaaaeaacqqGubavdaWgaaWcbaGaee4zaCMaeeiAaGgabeaaaOqaaiabbAfawnaaBaaaleaacqqGNbWzaeqaaaaaaOGaayjkaiaawMcaaaGaay5waiaaw2faaiabbccaGiabg2da9iabbUealnaaBaaaleaacqqG4baEcqqGNbWzcqqGjbqsaeqaaaaa@6998@

It can be shown [34] that the solutions of the proposed discrete Single-Delay Model for I and G are positive and bounded for all times, and that their time-derivatives are also bounded for all times. Further, the model admits the single (positive-concentration) equilibrium point (G_b_, I_b_). The system is also asymptotically locally stable around its equilibrium point. Parameters G* and V_i _are set respectively to 9 mM and 0.25 L (kgBW)^-1^, so that the set of free parameters of the final model to be estimated is {V_g_, I_ΔG_, τ_g_, K_xgI_, K_xi_, γ}.

**Figure 2:**
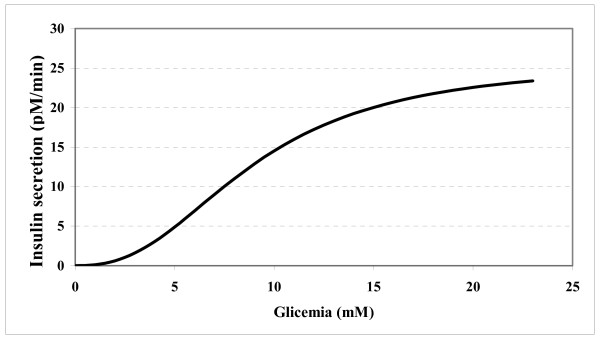
**Second-phase pancreatic insulin secretion**. Insulin secretion versus plasma glucose concentrations, as computed from the average values of the discrete SDM parameters.

Figure 2 shows the shape of the dynamics of insulin release predicted by the model, resulting from the average parameter values estimated on the 40 subjects.

### The Minimal Model

The two equations of the standard Minimal Model are written as follows:

dG(t)dt=−[b1+X(t)]G(t)+b1Gb,G(0)=b0
 MathType@MTEF@5@5@+=feaafiart1ev1aaatCvAUfKttLearuWrP9MDH5MBPbIqV92AaeXatLxBI9gBaebbnrfifHhDYfgasaacH8akY=wiFfYdH8Gipec8Eeeu0xXdbba9frFj0=OqFfea0dXdd9vqai=hGuQ8kuc9pgc9s8qqaq=dirpe0xb9q8qiLsFr0=vr0=vr0dc8meaabaqaciaacaGaaeqabaqabeGadaaakeaafaqabeqacaaabaWaaSaaaeaacqqGKbazcqqGhbWrcqGGOaakcqqG0baDcqGGPaqkaeaacqqGKbazcqqG0baDaaGaeyypa0JaeyOeI0YaamWaaeaacqqGIbGydaWgaaWcbaGaeGymaedabeaakiabgUcaRiabbIfayjabcIcaOiabbsha0jabcMcaPaGaay5waiaaw2faaiabbEeahjabcIcaOiabbsha0jabcMcaPiabgUcaRiabbkgaInaaBaaaleaacqaIXaqmaeqaaOGaee4raC0aaSbaaSqaaiabbkgaIbqabaGccqGGSaalaeaacqqGhbWrcqqGOaakcqqGWaamcqqGPaqkiiaacqWF9aqpcqqGIbGydaWgaaWcbaGaeeimaadabeaaaaaaaa@52AE@

dX(t)dt=−b2X(t)+b3(I(t)−Ib),I(0)=Ib
 MathType@MTEF@5@5@+=feaafiart1ev1aaatCvAUfKttLearuWrP9MDH5MBPbIqV92AaeXatLxBI9gBaebbnrfifHhDYfgasaacH8akY=wiFfYdH8Gipec8Eeeu0xXdbba9frFj0=OqFfea0dXdd9vqai=hGuQ8kuc9pgc9s8qqaq=dirpe0xb9q8qiLsFr0=vr0=vr0dc8meaabaqaciaacaGaaeqabaqabeGadaaakeaafaqabeqacaaabaWaaSaaaeaacqqGKbazcqqGybawcqGGOaakcqqG0baDcqGGPaqkaeaacqqGKbazcqqG0baDaaGaeyypa0JaeyOeI0IaeeOyai2aaSbaaSqaaiabikdaYaqabaGccqqGybawcqGGOaakcqqG0baDcqGGPaqkcqGHRaWkcqqGIbGydaWgaaWcbaGaeG4mamdabeaakiabcIcaOiabbMeajjabcIcaOiabbsha0jabcMcaPiabgkHiTiabbMeajnaaBaaaleaacqqGIbGyaeqaaOGaeiykaKIaeiilaWcabaGaeeysaKKaeeikaGIaeeimaaJaeeykaKcccaGae8xpa0JaeeysaK0aaSbaaSqaaiabbkgaIbqabaaaaaaa@52DF@

The symbols are defined in Table 4.

**Table 4:** Definition of the symbols in the Minimal Model

**Symbol**	**Units**	**Definition**
t	[min]	time after the glucose bolus
G(t)	[mM]	blood glucose concentration at time t
X(t)	[min^-1^]	auxiliary function representing insulin-excitable tissue glucose uptake activity, proportional to insulin concentration in a "distant" compartment
G_b_	[mM]	subject's basal (pre-injection) glycemia
I_b_	[pM]	subject's basal (pre-injection) insulinemia
b_0_	[mM]	theoretical glycemia at time 0 after the instantaneous glucose bolus
b_1_	[min^-1^]	glucose mass action rate constant, i.e. the insulin-independent rate constant of tissue glucose uptake, "glucose effectiveness"
b_2_	[min^-1^]	rate constant expressing the spontaneous decrease of tissue glucose uptake ability
b_3_	[min^-2 ^pM^-1^]	insulin-dependent increase in tissue glucose uptake ability, per unit of insulin concentration excess over baseline insulin
S_I _(b_3_/b_2_)	[min^-1 ^pM^-1^]	insulin sensitivity index and represents the capability of tissue to uptake circulating plasma glucose

The Minimal Model [10] describes the time-course of glucose plasma concentrations, depending upon insulin concentrations and makes use of the variable X, representing the 'Insulin activity in a remote compartment'. While in later years different versions of the Minimal Model appeared [35,36], the original formulation reported above is most widely employed, even in recent research applications [37-44].

### Statistical Methods

For each subject the four alternative models (A, B, C, D, described in table 2) have been fitted to glucose and insulin plasma concentrations by Generalized Least Squares (GLS, described in Appendix 1) in order to obtain individual regression parameters. All observations on glucose and insulin have been considered in the estimation procedure except for the basal levels. Coefficients of variation (CV) for glucose and insulin were estimated with phase 2 of the GLS algorithm, whereas single-subject CVs for the model parameter estimates were derived from the corresponding variances, obtained from the diagonal elements of the estimated asymptotic variance-covariance matrix of the GLS estimators. The individual-specific regression parameters were then used for population inference.

For the Minimal Model, fitting was performed by means of a Weighted Least Squares (WLS) estimation procedure, considering as weights the inverses of the squares of the expectations and as coefficients of variation 1.5% for glucose and 7% for insulin [9]. Observations on glucose before 8 minutes from the bolus injection, as well as observations on insulin before the first peak were disregarded, as suggested by the proposing Authors [9,10]. A BFGS quasi-Newton algorithm was used for all optimizations [45]. A-posteriori model identifiability was determined by computing the asymptotic coefficient of variation (CV) for the free model parameters: a CV smaller than 52% translates into a standard error of the parameter smaller than 1/1.96 of its corresponding point estimate and into an asymptotic confidence region of the parameter not including zero.

In order to compare the two models under the same statistical estimation scheme, the Minimal Model was also fitted to observed data points using the same GLS algorithm employed for the SDM.

## Results

### Delay Model Selection

Each delay model (A, B, C and D) was fitted on data from each one of the experimental subjects and the Akaike Information Criterion (AIC) was computed. Six paired t-tests were performed (A vs. B, A vs. C, A vs. D, B vs. D, C vs. D and B vs. C). Model A had the lowest average on the individual AIC's. All tests were conducted at a level alpha of 0.05 and differences were found to be statistically significant (A vs. B, P < 0.001; A vs. C, P < 0.001; A vs. D, P < 0.001; B vs. D, P = 0.036; C vs. D, P = 0.002), except for the comparison B vs. C, which was found to be non-significant (P = 0.191). The best model under the AIC criterion was therefore model A, which performed significantly better than either model B or C, which in turn performed significantly better than model D.

### Model Parameter Estimates

For the discrete SDM the parameter coefficients of variation were derived for each subject from the asymptotic results for GLS estimators. Coefficients of variation for all parameters in all subjects were found to be smaller than 52%, except: for parameter τ_g_, which in 5 subjects was estimated to about zero, producing therefore a large CV, and which otherwise exhibited a large CV in 13 other subjects; for parameter γ, in those 3 subjects for whom it was estimated at a value less than 1 as well as for another single subject; and for parameter K_xi _in 2 subjects.

For the MM, the corresponding standard errors and coefficients of variation (for each parameter and for each subject) were computed by applying standard results for weighted least square estimators, where the coefficients of variation for glucose and insulin were set respectively to 1.5% and 7%. Parameters of the MM were also estimated by means of the same GLS procedure employed for the SDM. However, since for all parameters and individuals the resulting confidence regions were as large as or larger than the corresponding WLS regions, only the more favorable results obtained by WLS were retained for comparison.

**Figure 3:**
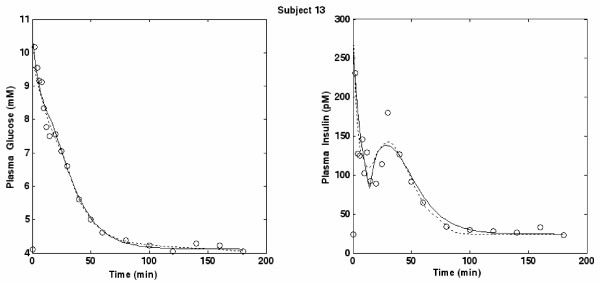
**Plot for Subject 13**. Glucose and Insulin (circles) concentrations versus time together with the predicted time-curves from the SDM (continuous lines) and the MM (dotted lines) for subject 13.

**Figure 4:**
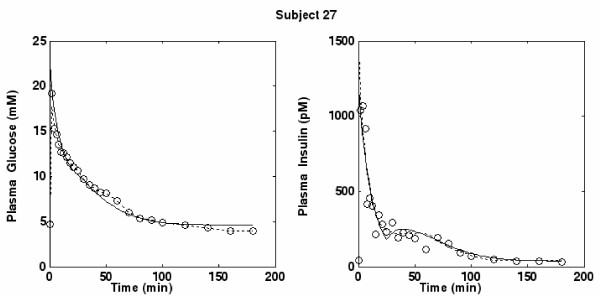
**Plot for Subject 27**. Glucose and Insulin (circles) concentrations versus time together with the predicted time-curves from the SDM (continuous lines) and the MM (dotted lines) for subject 27.

**Figure 5:**
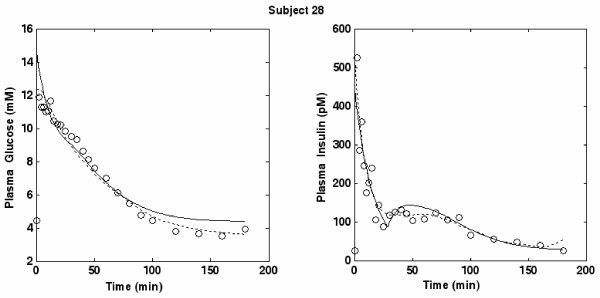
**Plot for Subject 28**. Glucose and Insulin (circles) concentrations versus time together with the predicted time-curves from the SDM (continuous lines) and the MM (dotted lines) for subject 28.

Figures 3, 4 and 5 portray three typical subjects with both insulin and glucose concentration observations, as well as predicted time courses based on the discrete SDM and the MM. In order to have a comparison curve for predicted insulin, the original Minimal Model for Insulin secretion [10], fitted by means of the original procedure described by Pacini [46], was employed. For subjects 13 and 27 (figures 3 and 4) the predicted curves are nearly superimposed. For subject 28 (Figure 5), while the MM curve seems closer to the points than that of the SDM, its predicted insulin concentrations are visibly increasing at the end of the observation period (and will be predicted to increase to extremely high levels within a few hours), instead of tending to the equilibrium value I_b_. This behavior is common to a few subjects (for subjects 23, 25 and 28 most evidently over 180 minutes) and is consistent with the theoretical results demonstrated in De Gaetano and Arino [31].

**Figure 6:**
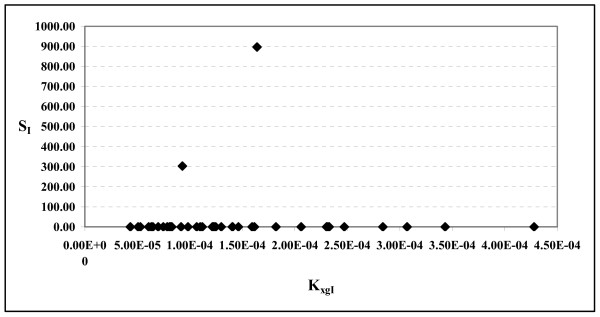
**S_I _versus K_xgI _in the whole sample**. Scatter plot between the Insulin Sensitivity (S_I_) derived from MM and the parameter K_xgI _over the whole sample of 40 subjects.

**Figure 7:**
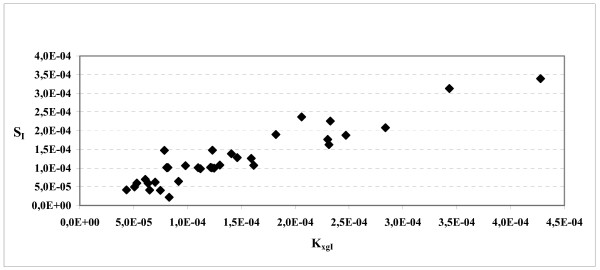
**S_I _versus K_xgI _in the reduced sample**. Scatter plot between the Insulin Sensitivity (S_I_) derived from MM and the parameter K_xgI _over the reduced sample of 35 subjects.

Figures 6 and 7 report the scatter plots between K_xgI _and S_I_. In the first figure all 40 subjects were considered, whereas for the second figure, 5 subjects were discarded: they were those subjects whose indices of insulin-sensitivity S_I _from the MM were either very small (less than 1.0 × 10^-5^) or very large (greater than 1.0 × 10^2^). In all these cases the coefficients of variation of S_I _were found to be very large, varying between 1545% and 2.36 × 10^9^%. If these extreme-S_I _subjects are not considered, the scatter plot of figure 7 shows a clear positive correlation between K_xgI _and S_I _(r = 0.93).

It has been demonstrated that the homeostasis model assessment insulin resistance index HOMA-IR (computed as the product of the fasting values of glucose, expressed as mM, and insulin, expressed as μU/mL, divided by the constant 22.5) [47-49], its reciprocal insulin sensitivity index 1/HOMA-IR [50], and the quantitative insulin sensitivity check index QUICKI [51] are useful surrogate indices of insulin resistance because of their high correlation with the index assessed by the euglycemic hyperinsulinemic clamp [11].

The insulin sensitivity index 1/HOMA-IR was therefore compared to the estimated S_I _and K_xgI _parameter values. Table 5 reports the correlation results. The upper part of the table reports results referred to the whole sample of 40 subjects, while the lower part of the table does not consider the 5 subjects for which the S_I _index could not be reliably computed. The correlation between 1/HOMA-IR and K_xgI _is about the same in the two analyses and is significant in both, whereas the correlation between 1/HOMA-IR and S_I _is positive and significant only in the reduced 35-subject sample.

**Table 5:** Correlation between 1/HOMA-IR and the two insulin-sensitivity indices K_xgI _and S_I_

		**K**_**xgI**_	**S**_**I**_
**1/HOMA-IR**	**Pearson Correlation**	0.588	-0.151
**full sample**	**Sig. (2-tailed)**	< 0.001	0.351
	**N**	40	40

			
		**K**_**xgI**_	**S**_**I**_

**1/HOMA-IR**	**Pearson Correlation**	0.599	0.569
**reduced sample**	**Sig. (2-tailed)**	< 0.001	< 0.001
	**N**	35	35

In order to evaluate the performance of the MM also under conditions of arbitrary stabilization of the parameter estimates, WLS data fitting with the Minimal Model was repeated when constraining parameters b_2 _and b_3_, setting their lower bounds to 10^-5 ^and 10^-7 ^respectively. The use of boundaries for parameter values in the optimization process leading to parameter estimation can be a legitimate procedure, especially when starting the optimization, in order to facilitate convergence of the sequence of estimates to the optimum. However, the optimum eventually reached must lie in the interior of the specified region of parameter space in order for it to be a local optimum and for the statistical properties of the resulting estimate to be maintained.

In the case where the optimum lies at one of the boundaries, the gradient of the loss function with respect to the parameter is not zero, the point is not an isolated local optimum and the properties of the considered estimator (Ordinary Least Square, Weighted Least Square or Maximum Likelihood) are lost.

In our case, when arbitrarily delimiting the MM parameters, we did frequently obtain optima at the boundary of the acceptance region. In this case, the predicted curves were as good as in the original 'unconstrained' MM analysis, but parameter estimates sometimes were found to be very different. With this latter procedure 7 subjects exhibited S_I_index values greater than 1 × 10^-2^; the correlation coefficient with the 1/HOMA-IR was 0.173 (P = 0.287) when all 40 subjects were considered and 0.396 (P = 0.023) when these 7 subjects were excluded.

Table 6 reports the sample means of the parameter estimates of the discrete SDM, whereas Table 7 reports the same results for the MM estimated with the standard WLS approach.

**Table 6:** Descriptive Statistics of the parameter estimates for the SDM on the whole sample.

**Sample parameter estimates: descriptive statistics**						
**Parameter**	**V**_**g**_	**I**_**Δ**_	**τ**_**g**_	**K**_**xgI**_	**k**_**xi**_	**γ**

**Mean**	0.152	41.791	19.271	1.43E-04	0.101	2.464
**SD**	0.050	20.637	12.156	8.7 E-05	0.079	0.875
**CV (%)**	32.66	49.38	63.08	60.93	78.00	35.53
**SE**	0.0079	3.2631	1.9220	1.38E-05	0.0124	0.1384
**CV (%)**	5.16	7.81	9.97	9.63	12.33	5.62
**min**	0.065	11.686	3.58E-37	4.34E-05	0.0314	0.736
**max**	0.292	90.90	60	4.28E-04	0.480	4.122

**Sample correlation matrix of the parameter estimates**						

	**V**_**g**_	**I**_**Δ**_	**τ**_**g**_	**K**_**xgI**_	**k**_**xi**_	**γ**

**V**_**g**_	1	0.248	0.044	-0.454	-0.353	0.136
**I**_**Δ**_		1	0.203	-0.529	0.059	0.117
**τ**_**g**_			1	-0.403	-0.383	-0.185
**K**_**xgI**_				1	0.552	-0.288
**k**_**xi**_					1	0.098
**γ**						1
σG2 MathType@MTEF@5@5@+=feaafiart1ev1aqatCvAUfKttLearuWrP9MDH5MBPbIqV92AaeXatLxBI9gBaebbnrfifHhDYfgasaacH8akY=wiFfYdH8Gipec8Eeeu0xXdbba9frFj0=OqFfea0dXdd9vqai=hGuQ8kuc9pgc9s8qqaq=dirpe0xb9q8qiLsFr0=vr0=vr0dc8meaabaqaciaacaGaaeqabaqabeGadaaakeaaiiqacqWFdpWCdaqhaaWcbaacbeGae43raCeabaGae4Nmaidaaaaa@30A7@	0.039	**CV**_**G**_	19.75%			
σI2 MathType@MTEF@5@5@+=feaafiart1ev1aqatCvAUfKttLearuWrP9MDH5MBPbIqV92AaeXatLxBI9gBaebbnrfifHhDYfgasaacH8akY=wiFfYdH8Gipec8Eeeu0xXdbba9frFj0=OqFfea0dXdd9vqai=hGuQ8kuc9pgc9s8qqaq=dirpe0xb9q8qiLsFr0=vr0=vr0dc8meaabaqaciaacaGaaeqabaqabeGadaaakeaaiiqacqWFdpWCdaqhaaWcbaacbeGae4xsaKeabaGae4Nmaidaaaaa@30AB@	0.099	**CV**_**I**_	31.46%			

**Table 7:** Descriptive Statistics of the parameter estimates from the WLS methods for the MM.

**Sample parameter estimates for the 40 Subjects**									
**Parameter**	**b**_**0**_	**b**_**1**_	**b**_**2**_	**b**_**3**_	**b**_**4**_	**b**_**5**_	**b**_**6**_	**b**_**7**_	**S**_**I**_

**Values**	13.415	0.016	0.061	6.59E-06	0.425	5.091	0.136	618.82	30.00
**SD**	2.605	0.016	0.107	1.11E-05	1.428	1.362	0.065	311.51	148.48
**CV (%)**	19.42	98.91	174.90	168.85	335.99	26.75	47.43	50.34	494.99
**SE**	0.407	0.003	0.017	1.74E-06	0.223	0.213	0.010	48.65	23.19
**CV (%)**	3.03	15.45	27.32	26.37	52.47	4.18	7.41	7.86	77.30

**Sample parameter estimates for the 35 Subjects**									

**Parameter**	**b**_**0**_	**b**_**1**_	**b**_**2**_	**b**_**3**_	**b**_**4**_	**b**_**5**_	**b**_**6**_	**b**_**7**_	**S**_**I**_

**Values**	13.251	0.013	0.066	7.49E-06	0.222	5.023	0.136	632.869	1.25E-04
**SD**	2.175	0.012	0.113	1.16E-05	0.372	1.357	0.064	319.523	7.40E-05
**CV (%)**	16.42	92.92	172.24	155.47	167.22	27.02	47.04	50.49	59.35
**SE**	0.340	0.002	0.018	1.82E-06	0.058	0.212	0.010	49.901	1.16E-05
**CV (%)**	2.56	14.51	26.90	24.28	26.12	4.22	7.35	7.88	9.27

**Sample correlation matrix of the parameter estimates for the 40 Subjects**									

	**b**_**0**_	**b**_**1**_	**b**_**2**_	**b**_**3**_	**b**_**4**_	**b**_**5**_	**b**_**6**_	**b**_**7**_	

**b**_**0**_	1	0.588	-0.264	-0.270	-0.224	0.194	0.023	0.073	
**b**_**1**_		1	-0.190	-0.199	0.118	0.289	0.091	0.051	
**b**_**2**_			1	0.960	-0.082	-0.165	0.147	-0.126	
**b**_**3**_				1	-0.081	-0.185	0.180	-0.209	
**b**_**4**_					1	0.506	-0.097	0.020	
**b**_**5**_						1	-0.184	0.301	
**b**_**6**_							1	0.140	
**b**_**7**_								1	

**Sample correlation matrix of the parameter estimates for the 35 Subjects**									

	**b**_**0**_	**b**_**1**_	**b**_**2**_	**b**_**3**_	**b**_**4**_	**b**_**5**_	**b**_**6**_	**b**_**7**_	

**b**_**0**_	1	0.410	-0.300	-0.314	0.151	0.386	-0.003	0.185	
**b**_**1**_		1	-0.155	-0.136	0.539	0.429	0.090	0.203	
**b**_**2**_			1	0.968	0.022	-0.145	0.151	-0.153	
**b**_**3**_				1	-0.011	-0.174	0.204	-0.249	
**b**_**4**_					1	0.694	0.247	0.487	
**b**_**5**_						1	-0.123	0.384	
**b**_**6**_							1	0.149	
**b**_**7**_								1	

It is of interest to note that K_xgI _and S_I_, which measure the same phenomenon, have the same theoretical definition and are computed in the same units, coincide very well in absolute numerical value when the 5 subjects discussed above are not considered (K_xgI _= 1.40 ×10^-4 ^min^-1^pM^-1 ^vs. S_I _= 1.25 ×10^-4 ^min^-1^pM^-1^). K_xgI _and S_I_, on the other hand, differ markedly if the whole sample is considered (K_xgI _= 1.43 ×10^-4 ^min^-1^pM^-1 ^vs. S_I _= 30 min^-1 ^pM^-1^).

Coefficients of variation for glucose and insulin, when considering the discrete SDM, were estimated by GLS to be respectively 19.8% and 31.5%. (for the MM, when adopting the GLS procedure, they were estimated to be respectively 17.5% and 30.9%). Although the estimated values are much larger than those reported in literature [9] (1.5% for glucose and 7% for insulin), they reflect both the variability due to measurement error and the variability due to actual oscillation of glucose and insulin concentrations in plasma. While these error estimates are rather large, they may be more realistic, in vivo, than simple estimates of the variance of repeated laboratory in-vitro measurements on the same sample.

## Discussion

The present work introduces a new model for the interpretation of glucose and insulin concentrations observed during an IVGTT. The model has been tested in a sample of "normal" subjects: these subjects' IVGTTs were selected from a larger group of available IVGTTs on the basis of normality of baseline Glycemia (< 7 mM) and 'normality' of BMI (< 30 Kg m^-2^).

### Presentation of the physiological assumptions underlying the discrete Single-Delay Model

The new model was chosen on the basis of the Akaike criterion from a group of four different two-compartment models: all models in the group included first-order insulin elimination kinetics, second-order insulin-dependent net glucose tissue uptake, a zero-order net hepatic glucose output, and progressively increasing but eventually saturating pancreatic insulin secretion in response to rising glucose concentrations. The differences among the four tested models were that, while one model included both an explicit delay in the action of circulating insulin on glucose transport, as well as a term for insulin-independent tissue glucose uptake, one model only included insulin delay, another model only included insulin-independent glucose uptake, and the final model included neither. This final model was chosen because, from a purely numerical point of view, neither the addition of a delay in the insulin action on glucose transport, nor the addition of an insulin-independent, first-order glucose elimination term appeared to improve the model fit to observations.

The delay in the glucose action on pancreatic response, expressed in the discrete SDM by the explicit term τ_g_, was found to be necessary if a second-phase insulin response was to produce an evident insulin concentration 'hump'. For this reason, this delay was included in all four models tested in the present work.

It is somewhat surprising that the best model among those studied does not require an explicit delay in insulin action on glucose transport, which had been expressed in the Minimal Model by the 'remote-compartment' insulin activity X [9]. Some reports had in fact indirectly substantiated [52,53] an anatomical basis for this delay: it should be kept in mind, however, that an actual delay in the cellular or molecular action of the hormone is not at all necessary in order to explain the commonly apparent delay in hormone effect, as judged by a perceptible decrease in glucose concentrations. In other words, even if the action of the hormone on its target is not retarded, its actual perceptible effect may well exhibit a delay. Thus a mathematical model of the system may correctly show a delayed effect of insulin even in the absence of an explicit term representing retarded action of the hormone. In any case, an explicit representation of this mechanism does not seem necessary to explain the observations in the present series.

Another difference with respect to commonly accepted concepts is the lack of a "glucose effectiveness term", i.e. of a first-order, insulin-independent tissue glucose uptake rate term. Except for the fact that it has become customary to see this term included in glucose/insulin models, there appears to be no physiological mechanism to support first-order glucose elimination from plasma, when exception is made of glycemias above the renal threshold and when diffusion into a different compartment is discounted. Tissues in the body (except for brain) do not take up glucose irrespective of insulin: brain glucose consumption is relatively constant, and is subsumed, for the purposes of the present model, in the constant net (hepatic) glucose output term. It must be emphasized that none of the subjects studied exhibited sustained, above-renal-threshold glycemias. It is therefore likely that, even if such a first-order mechanism were indeed present, its explicit representation did not prove indispensable for the acceptable fitting of the present data series. In future analyses it may be necessary to reintroduce insulin action delay or first order insulin-independent glucose uptake or both.

### Remarks on decoupled fitting versus single-pass fitting of data points

It is of interest to comment on a widespread conception that interpolated observed data, used in place of theoretically reconstructed curves, are a reliable approximation of the true signal for the purpose of parameter estimation. This approach has been used, for instance, in the original 'decoupling' method of parameter estimation for the MM [46], which we will use simply as an example for illustrating the present remarks.

The strategy of fitting one state variable at a time (while assuming the linearly interpolated, noisy observations of the other state variable to provide the true input function) decouples the regulatory system: the expected feedback effect, of the state variable being fitted onto the other state variable, is disregarded. It happens thus that the estimated parameters are optimal in predicting the observed glucose assuming the erratic observed insulin as the true value of the insulin concentration, but are far from optimal when the expected glucose determines the expected insulin and is then determined by it in its turn. This separate fitting strategy produces sets of estimated parameters such that the expected time course of glucose using the expected time course of insulin as input may differ markedly from both the actual glucose observations and from the expected glucose obtained using the noisy insulin observations as input. In other words, the separate fitting strategy produces parameter values which do not make model predictions of glucose and insulin consistent with each other.

**Figure 8:**
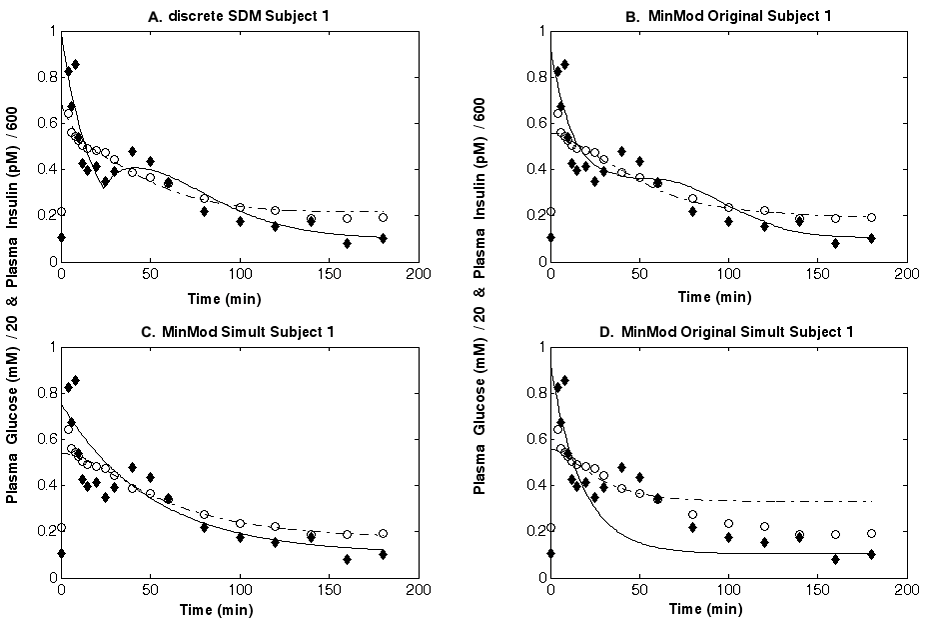
**Comparison between different methods of data fitting**. Each figure reports Glucose (white circles) and Insulin (black diamonds) observed concentrations versus time, together with the predicted time-curves (dashed line for Glucose and continuous line for Insulin) using four different methods: the discrete Single Delay Model (figure 8.a); the Minimal Model with its traditional two-pass 'decoupling' estimation method (figure 8.b); the Minimal Model when all parameters are fitted simultaneously (figure 8.c); the Minimal Model when global system behavior (interacting glycemias and insulinemias) is reconstructed from separately estimated (two-pass) parameters (figure 8.d).

In order to clarify the statement above and to show that the concerns raised are far from purely philosophical, Figure 8 shows four sets of model-reconstructed curves associated with the same data set. In figure 8.a the observed points are fitted with the SDM (one-pass fitting, minimizing errors in glycemias and insulinemias simultaneously) and the resulting SDM-predicted time courses are superimposed. The fitting, with six parameters, is reasonably good and a second-phase insulin secretion "hump" is clearly visible.

In figure 8.b the observed points are fitted with the 'decoupling' Minimal Model based on three equations [46] (two-pass fitting, using interpolated insulinemia as input for the fit of glycemias, then interpolated glycemia as input for the fit of insulinemia). The predicted curves lie close to the observations (in this set-up eight parameters are free) and second-phase insulin secretion is readily apparent. In figure 8.c the observations are fitted with the same three MM equations, this time using a simultaneous, one-pass procedure. In this way, glycemias and insulinemias are simultaneously predicted from the model and parameters are adjusted to provide the best overall weighted fit. While the predicted curves pass through the observed points, no second-phase insulin secretion hump is visible. In fact, best estimates for the simultaneous three-equation MM parameters never produced a visible second-phase insulin secretion hump in any one of the 40 subjects from the present series. Finally, figure 8.d shows the original observations and the curves obtained when using simultaneously the classical MM parameter estimates. In other words, in figure 8.d the same parameter values obtained in the classical 'decoupling' MM fit of figure 8.b are employed. This time, however, instead of using the recorded noisy observations to provide feedback regulation, the actual predictions of the model are used, so that predicted glycemia influences the prediction of insulinemia and vice-versa. It can be appreciated how, in this case, predictions fail to approximate observations.

If it is required that the identified model be consistent, i.e. that the functional form of the model, together with the estimated parameter values, reproduce the dynamics actually observed, then decoupling the feedback and estimating separately its two arms provides misleading results: while it would seem that the fit is good (Figure 8.b), such good fit actually relies on the specific realization of a chance occurrence of errors in the observations. In this way parameters are obtained which can apparently reproduce features (like in this case the second-phase insulin secretion hump), but can do so only by exploiting that experiment's specific observation errors. When these same parameters are used to model the interaction of predicted glycemias and insulinemias on each other (as in figure 8.d), no such features appear and indeed, even actual data fit is very poor.

### Comparison of Insulin Sensitivity determinations from the SDM and the MM

The possibility to reliably estimate an index of insulin-sensitivity is essential to any model which aims at being useful to diabetologists. In the following, we discuss the comparison between the newly-introduced Discrete Single Delay Model, and the Minimal Model in its (to date) uncontroversial formulation, i.e. considering only the two equations (4) and (5), fitted using interpolated insulinemias as forcing function, from which the insulin sensitivity index S_I _is computed. This is the 'standard' MM, being currently used in many experimental applications [37-44].

By applying the same definition of the Insulin Sensitivity Index to both the discrete SDM and the standard MM, we obtain quantities (the K_xgI _and the S_I_), which have the same units of measurement and, over the restricted subject sample, approximately the same average value and a correlation coefficient of 0.93.

One evident difference between the performances of the discrete SDM and the MM over the 40 subjects considered in this series relates to the stability of estimation, in particular with respect to the insulin sensitivity indices (K_xgI _for the SDM and S_I _for the MM). Whereas in every one of the 40 subjects considered, the estimate of K_xgI _had a coefficient of variation smaller than 52% (i.e. its 95% asymptotic confidence region excluded zero), in 20 out of 40 'normal' subjects the S_I _did not result significantly different from zero.

Correlation between the S_I_ and the K_xgI _was poor when considering all 40 subjects, very good when excluding five subjects whose S_I _was either very large or very small. Average values of S_I _varied by five orders of magnitude, and correlation with 1/HOMA-IR dropped, when going from the restricted 35-subject sample to the full 40-subject sample. Average values of the K_xgI _and correlation of the K_xgI _with 1/HOMA-IR were very similar when using either the full sample or the restricted sample.

Besides the insulin-sensitivity index, all other model parameters were generally identifiable with the discrete SDM and often not identifiable with the MM, pointing to the fact that the MM appears overparametrized with respect to the information available from the standard IVGTT.

It is worth to point out that there is a clear difference between stability and accuracy. In this respect, the result which should be considered is, in our opinion, the correlation with the 1/HOMA-IR: when parameter estimates with the MM are numerically stable (when boundary parameter estimates are avoided and in those cases where extreme values are not produced, i.e. the 35-subject reduced sample), then the MM results correlate well with the other indices (SDM, 1/HOMA-IR), and we should conclude that in this case the three methods deliver more or less accurate estimation of the actual insulin sensitivity of the subjects. When numerical problems in the MM estimation procedure occur (i.e. when considering also the 5 subjects with estimation problems), this correlation, between MM on one hand and SDM or 1/HOMA-IR on the other, is lost (while correlation between SDM and 1/HOMA-IR is always maintained, whether with 40 or with 35 subjects) and extreme S_I _coefficient values are produced. In this case, it would probably be reasonable to think that the two other methods, agreeing with each other and producing plausible numerical estimates, would be more accurate than the MM.

The five poorly identified S_I_'s had been singled out as being non-significantly different from zero and either extremely small or extremely large; but in fact the 20 poorly identified S_I_'s (with CV > 52%) were distributed over the entire observed S_I _range: this would contradict the simplistic postulation that only those S_I_'s are unidentifiable which are too small to be measured (being so low that their confidence interval would include the zero value assuming a constant variance throughout the range), hence that typically the unidentifiable S_I_'s correspond to high degrees of insulin resistance.

The five subjects referred to above (subjects 5, 16, 32, 36, 38) had no problems with the SDM (K_xgI_'s estimated at 1.07 × 10^-4^, 9.28 × 10^-5^, 1.64 × 10^-4^, 1.41 × 10^-4 ^and 3.07 × 10^-4 ^respectively), while their S_I _estimates under the MM (1.53 × 10^-6^, 3.03 × 10^+2^, 8.97 × 10^+2^, 1.00 × 10^-12 ^and 1.51 × 10^-12^) were extreme because, in order to accurately fit the observations, the values of either the b_2 _coefficient or the b_3 _coefficient were set essentially to zero (subj 5, b_3_= 5.37 × 10^-8^; subj 16, b_2 _= 1.86 × 10^-9^; subj 32, b_2_= 1.14 × 10^-9^; subj 36, b_3 _= 6.35 × 10^-14^; subj 38, b_2_= 6.63 × 10^-14^).

This happens because, in these five subjects in particular, observed insulinemias display an erratic behavior. Since the MM does not use a model for insulinemia, but uses interpolated (error-containing) observations on insulinemia to drive the glycemia model, parameters get estimated so as to explain observed glycemias on the basis of erratic insulinemias, and sometimes these parameters will be off-scale. The same may occur, due to relative overparametrization, if it is the glycemias which exhibit correlated errors or large oscillations, even in the presence of smoothly varying observed insulinemias.

The occurrence of "zero-S_I_" values (S_I _values with very low point estimation) has long been a recognized problem with the MM. For instance, in 1997, Ni et al. [54] affirmed that "... the occurrence of S_I _values indistinguishable from zero ("zero-S_I_") is not negligible in large clinical studies". This was supposed to be due to inaccurate one-compartment modelling of the glucose kinetics, and to be resolved by the use of a more complex two-compartment model (which would on the other hand have introduced more parameters in the estimation process). In the present series of non-obese subjects, while estimation with the MM gave rise to 3 out of 40 zero-S_I _cases, the S_I _was in fact not significantly different from zero in half of the subjects, while the SDM produced insulin sensitivity coefficients K_xgI _which were significantly different from zero in all subjects, (with a minimum K_xgI _of 4.34 × 10^-5^). It is therefore possible that overparametrization of the MM plays a greater role than the level of approximation (with a single rather than a double compartment for glucose) in the production of "zero-S_I_" estimates.

We finally note that the I_ΔG _parameter from the SDM has the same meaning as the dynamic responsivity index Φ_d _used by Campioni et al. [55] to characterize the secretion rate of insulin from the promptly releasable pool (assuming it proportional to the actual glucose concentration reached).

## Conclusion

The SDM has been designed for simultaneous fitting to glucose and insulin concentrations, and has been proven to have mathematically consistent solutions, admitting the fasting state as its single equilibrium point and converging back to it from the perturbed state. The sigmoidal shape of pancreatic insulin secretion in response to increasing glucose concentrations agrees with plausible physiology, since pancreatic ability to secrete insulin is limited.

In the present work it has been shown that, in 20 out of 40 healthy volunteers, while the standard Minimal Model fails to assess reliably the S_I _index, the SDM provides a precise estimate of insulin sensitivity. The present work therefore shows that the statistical, mathematical and physiological design features of the SDM actually translate, when the model is applied to data, into meaningful, robust estimates of insulin sensitivity from the standard IVGTT.

Future work in the evaluation of this model will entail testing it in samples of subjects with high prevalence of insulin resistance. Free software for fitting the SDM to a set of IVGTT data is available from the webpage of the CNR IASI Biomathematics Lab [56].

## Appendix

To obtain subject-specific model parameters and population estimates on the SDM the GLS method was used. GLS is a two-stage method:

(1) at first individual estimates βi∗
 MathType@MTEF@5@5@+=feaafiart1ev1aqatCvAUfKttLearuWrP9MDH5MBPbIqV92AaeXatLxBI9gBaebbnrfifHhDYfgasaacH8akY=wiFfYdH8Gipec8Eeeu0xXdbba9frFj0=OqFfea0dXdd9vqai=hGuQ8kuc9pgc9s8qqaq=dirpe0xb9q8qiLsFr0=vr0=vr0dc8meaabaqaciaacaGaaeqabaqabeGadaaakeaacqaHYoGydaqhaaWcbaGaeeyAaKgabaGaey4fIOcaaaaa@30C3@ for each subject i (i = 1,...,40) are obtained;

(2) then the estimates βi∗
 MathType@MTEF@5@5@+=feaafiart1ev1aqatCvAUfKttLearuWrP9MDH5MBPbIqV92AaeXatLxBI9gBaebbnrfifHhDYfgasaacH8akY=wiFfYdH8Gipec8Eeeu0xXdbba9frFj0=OqFfea0dXdd9vqai=hGuQ8kuc9pgc9s8qqaq=dirpe0xb9q8qiLsFr0=vr0=vr0dc8meaabaqaciaacaGaaeqabaqabeGadaaakeaacqaHYoGydaqhaaWcbaGaeeyAaKgabaGaey4fIOcaaaaa@30C3@ are used to construct the population estimates.

When observations are taken at different times from several subjects, it is important to take into account in the modeling procedure two sources of variability: random variation among measurements within a given individual and random variation among individuals. To accommodate these two different variance components a hierarchical statistical model was built:

### Stage 1 (intra-individual variation)

given the model:

y_i _= f_i_(β_i_) + e_i_

E(e_i_|β_i_) = 0 Cov(e_i_|β_i_) = R(β_i_,ξ)

where E(y_i_) = f_i _with f_i _representing the numerical solution of SDM for subject i, the variability within subject i is expressed by means of the functional form of R(β_i_,ξ), where the additional intra-individual covariance parameter vector ξ is the same across the individuals. Denoting with G and I respectively the state variable Glucose and the state variable Insulin, the variance-covariance matrix R in the present application has the structure of a block-diagonal matrix:

Ri=[RG00RI]
 MathType@MTEF@5@5@+=feaafiart1ev1aqatCvAUfKttLearuWrP9MDH5MBPbIqV92AaeXatLxBI9gBaebbnrfifHhDYfgasaacH8akY=wiFfYdH8Gipec8Eeeu0xXdbba9frFj0=OqFfea0dXdd9vqai=hGuQ8kuc9pgc9s8qqaq=dirpe0xb9q8qiLsFr0=vr0=vr0dc8meaabaqaciaacaGaaeqabaqabeGadaaakeaacqqGsbGudaWgaaWcbaGaeeyAaKgabeaakiabg2da9maadmaabaqbaeqabiGaaaqaaiabbkfasnaaBaaaleaacqqGhbWraeqaaaGcbaGaeGimaadabaGaeGimaadabaGaeeOuai1aaSbaaSqaaiabbMeajbqabaaaaaGccaGLBbGaayzxaaaaaa@393B@

where

Ri=[σG2fiG2(βi,ti1)...0.........0...σI2fiI2(βi,tiniI)].
 MathType@MTEF@5@5@+=feaafiart1ev1aaatCvAUfKttLearuWrP9MDH5MBPbIqV92AaeXatLxBI9gBaebbnrfifHhDYfgasaacH8akY=wiFfYdH8Gipec8Eeeu0xXdbba9frFj0=OqFfea0dXdd9vqai=hGuQ8kuc9pgc9s8qqaq=dirpe0xb9q8qiLsFr0=vr0=vr0dc8meaabaqaciaacaGaaeqabaqabeGadaaakeaacqqGsbGudaWgaaWcbaGaeeyAaKgabeaakiabg2da9maadmaabaqbaeqabmWaaaqaaiabeo8aZnaaDaaaleaacqqGhbWraeaacqaIYaGmaaGccqqGMbGzdaqhaaWcbaGaeeyAaKMaee4raCeabaGaeGOmaidaaOGaeiikaGIaeqOSdi2aaSbaaSqaaiabbMgaPbqabaGccqGGSaalcqqG0baDdaWgaaWcbaGaeeyAaKMaeGymaedabeaakiabcMcaPaqaaiabc6caUiabc6caUiabc6caUaqaaiabicdaWaqaaiabc6caUiabc6caUiabc6caUaqaaiabc6caUiabc6caUiabc6caUaqaaiabc6caUiabc6caUiabc6caUaqaaiabicdaWaqaaiabc6caUiabc6caUiabc6caUaqaaiabeo8aZnaaDaaaleaacqqGjbqsaeaacqaIYaGmaaGccqqGMbGzdaqhaaWcbaGaeeyAaKMaeeysaKeabaGaeGOmaidaaOGaeiikaGIaeqOSdi2aaSbaaSqaaiabbMgaPbqabaGccqGGSaalcqqG0baDdaWgaaWcbaGaeeyAaKMaeeOBa42aaSbaaWqaaiabbMgaPjabbMeajbqabaaaleqaaOGaeiykaKcaaaGaay5waiaaw2faaiabc6caUaaa@6AEC@

The parameters σG2
 MathType@MTEF@5@5@+=feaafiart1ev1aqatCvAUfKttLearuWrP9MDH5MBPbIqV92AaeXatLxBI9gBaebbnrfifHhDYfgasaacH8akY=wiFfYdH8Gipec8Eeeu0xXdbba9frFj0=OqFfea0dXdd9vqai=hGuQ8kuc9pgc9s8qqaq=dirpe0xb9q8qiLsFr0=vr0=vr0dc8meaabaqaciaacaGaaeqabaqabeGadaaakeaacqaHdpWCdaqhaaWcbaGaee4raCeabaGaeGOmaidaaaaa@30A4@ and σI2
 MathType@MTEF@5@5@+=feaafiart1ev1aqatCvAUfKttLearuWrP9MDH5MBPbIqV92AaeXatLxBI9gBaebbnrfifHhDYfgasaacH8akY=wiFfYdH8Gipec8Eeeu0xXdbba9frFj0=OqFfea0dXdd9vqai=hGuQ8kuc9pgc9s8qqaq=dirpe0xb9q8qiLsFr0=vr0=vr0dc8meaabaqaciaacaGaaeqabaqabeGadaaakeaacqaHdpWCdaqhaaWcbaGaeeysaKeabaGaeGOmaidaaaaa@30A8@, which have to be estimated, are the squares of the coefficients of variation respectively for glucose and insulin.

### Stage 2 (inter-individual variation)

In the second stage of the hierarchical model the variation among individuals (due for example to gender, age, treatment group or simply to biological variability among different individuals), is taken into account by means of a statistical model for the subject structural parameters β_i_. In this work the simplest case of a linear model has been considered:

β_i _= β + b_i_, b_i_~N(0, D)

where β is the vector of the fixed effects or the vector of the population parameters, whereas b_i _is the vector of the random effects for the i-th individual.

The Standard Two Stage method (STS) proceeds according to the following scheme:

#### STAGE 1

(1) In m separate estimation procedures (where m is the total number of subjects), obtain preliminary estimates βi(p)
 MathType@MTEF@5@5@+=feaafiart1ev1aqatCvAUfKttLearuWrP9MDH5MBPbIqV92AaeXatLxBI9gBaebbnrfifHhDYfgasaacH8akY=wiFfYdH8Gipec8Eeeu0xXdbba9frFj0=OqFfea0dXdd9vqai=hGuQ8kuc9pgc9s8qqaq=dirpe0xb9q8qiLsFr0=vr0=vr0dc8meaabaqaciaacaGaaeqabaqabeGadaaakeaacqaHYoGydaqhaaWcbaGaeeyAaKgabaGaeiikaGIaeeiCaaNaeiykaKcaaaaa@32ED@ for each individual i, i = 1,..., m (m = 40).

(2) Use residuals from these preliminary fits to estimate ξ=(σG2, σI2)
 MathType@MTEF@5@5@+=feaafiart1ev1aqatCvAUfKttLearuWrP9MDH5MBPbIqV92AaeXatLxBI9gBaebbnrfifHhDYfgasaacH8akY=wiFfYdH8Gipec8Eeeu0xXdbba9frFj0=OqFfea0dXdd9vqai=hGuQ8kuc9pgc9s8qqaq=dirpe0xb9q8qiLsFr0=vr0=vr0dc8meaabaqaciaacaGaaeqabaqabeGadaaakeaacqaH+oaEcqGH9aqpcqGGOaakcqaHdpWCdaqhaaWcbaGaee4raCeabaGaeGOmaidaaOGaeiilaWIaeeiiaaIaeq4Wdm3aa0baaSqaaiabbMeajbqaaiabikdaYaaakiabcMcaPaaa@3AD5@ minimizing the following function:

PL=∑i=1mlog⁡|Ri(βi(p),ξ)|+[yi−fi(βi(p))]TRi−1(βi(p),ξ)[yi−fi(βi(p))]
 MathType@MTEF@5@5@+=feaafiart1ev1aaatCvAUfKttLearuWrP9MDH5MBPbIqV92AaeXatLxBI9gBaebbnrfifHhDYfgasaacH8akY=wiFfYdH8Gipec8Eeeu0xXdbba9frFj0=OqFfea0dXdd9vqai=hGuQ8kuc9pgc9s8qqaq=dirpe0xb9q8qiLsFr0=vr0=vr0dc8meaabaqaciaacaGaaeqabaqabeGadaaakeaacqqGqbaucqqGmbatcqGH9aqpdaaeWbqaaiGbcYgaSjabc+gaVjabcEgaNnaaemaabaGaeeOuai1aaSbaaSqaaiabbMgaPbqabaGccqGGOaakcqaHYoGydaqhaaWcbaGaeeyAaKgabaGaeiikaGIaeeiCaaNaeiykaKcaaOGaeiilaWIaeqOVdGNaeiykaKcacaGLhWUaayjcSdGaey4kaSYaamWaaeaacqqG5bqEdaWgaaWcbaGaeeyAaKgabeaakiabgkHiTiabbAgaMnaaBaaaleaacqqGPbqAaeqaaOGaeiikaGIaeqOSdi2aa0baaSqaaiabbMgaPbqaaiabcIcaOiabbchaWjabcMcaPaaakiabcMcaPaGaay5waiaaw2faamaaCaaaleqabaGaeeivaqfaaOGaeeOuai1aa0baaSqaaiabbMgaPbqaaiabgkHiTiabigdaXaaakiabcIcaOiabek7aInaaDaaaleaacqqGPbqAaeaacqGGOaakcqqGWbaCcqGGPaqkaaGccqGGSaalcqaH+oaEcqGGPaqkdaWadaqaaiabbMha5naaBaaaleaacqqGPbqAaeqaaOGaeyOeI0IaeeOzay2aaSbaaSqaaiabbMgaPbqabaGccqGGOaakcqaHYoGydaqhaaWcbaGaeeyAaKgabaGaeiikaGIaeeiCaaNaeiykaKcaaOGaeiykaKcacaGLBbGaayzxaaaaleaacqqGPbqAcqGH9aqpcqaIXaqmaeaacqqGTbqBa0GaeyyeIuoaaaa@7E8C@

(3) Form estimated weight matrices based on the estimated parameters ξ^
 MathType@MTEF@5@5@+=feaafiart1ev1aqatCvAUfKttLearuWrP9MDH5MBPbIqV92AaeXatLxBI9gBaebbnrfifHhDYfgasaacH8akY=wiFfYdH8Gipec8Eeeu0xXdbba9frFj0=OqFfea0dXdd9vqai=hGuQ8kuc9pgc9s8qqaq=dirpe0xb9q8qiLsFr0=vr0=vr0dc8meaabaqaciaacaGaaeqabaqabeGadaaakeaacuaH+oaEgaqcaaaa@2E80@ and βi(p)
 MathType@MTEF@5@5@+=feaafiart1ev1aqatCvAUfKttLearuWrP9MDH5MBPbIqV92AaeXatLxBI9gBaebbnrfifHhDYfgasaacH8akY=wiFfYdH8Gipec8Eeeu0xXdbba9frFj0=OqFfea0dXdd9vqai=hGuQ8kuc9pgc9s8qqaq=dirpe0xb9q8qiLsFr0=vr0=vr0dc8meaabaqaciaacaGaaeqabaqabeGadaaakeaacqaHYoGydaqhaaWcbaGaeeyAaKgabaGaeiikaGIaeeiCaaNaeiykaKcaaaaa@32ED@:

R^i(βi(p),ξ^)
 MathType@MTEF@5@5@+=feaafiart1ev1aqatCvAUfKttLearuWrP9MDH5MBPbIqV92AaeXatLxBI9gBaebbnrfifHhDYfgasaacH8akY=wiFfYdH8Gipec8Eeeu0xXdbba9frFj0=OqFfea0dXdd9vqai=hGuQ8kuc9pgc9s8qqaq=dirpe0xb9q8qiLsFr0=vr0=vr0dc8meaabaqaciaacaGaaeqabaqabeGadaaakeaacuqGsbGugaqcamaaBaaaleaacqqGPbqAaeqaaOGaeeikaGIaeqOSdi2aa0baaSqaaiabbMgaPbqaaiabcIcaOiabbchaWjabcMcaPaaakiabcYcaSiqbe67a4zaajaGaeiykaKcaaa@3A25@

(4) Using the estimated weight matrices from step (3), re-estimate the β_i _by means of m minimizations: for each individual i, i = 1,... m minimize the following quantity

[yi−fi(βi)]TR−1(βi(p),ξ^)[yi−fi(βi)]
 MathType@MTEF@5@5@+=feaafiart1ev1aqatCvAUfKttLearuWrP9MDH5MBPbIqV92AaeXatLxBI9gBaebbnrfifHhDYfgasaacH8akY=wiFfYdH8Gipec8Eeeu0xXdbba9frFj0=OqFfea0dXdd9vqai=hGuQ8kuc9pgc9s8qqaq=dirpe0xb9q8qiLsFr0=vr0=vr0dc8meaabaqaciaacaGaaeqabaqabeGadaaakeaacqGGBbWwcqqG5bqEdaWgaaWcbaGaeeyAaKgabeaakiabgkHiTiabbAgaMnaaBaaaleaacqqGPbqAaeqaaOGaeiikaGIaeqOSdi2aaSbaaSqaaiabbMgaPbqabaGccqGGPaqkcqGGDbqxdaahaaWcbeqaaiabbsfaubaakiabbkfasnaaCaaaleqabaGaeyOeI0IaeGymaedaaOGaeiikaGIaeqOSdi2aa0baaSqaaiabbMgaPbqaaiabcIcaOiabbchaWjabcMcaPaaakiabcYcaSiqbe67a4zaajaGaeiykaKIaei4waSLaeeyEaK3aaSbaaSqaaiabbMgaPbqabaGccqGHsislcqqGMbGzdaWgaaWcbaGaeeyAaKgabeaakiabcIcaOiabek7aInaaBaaaleaacqqGPbqAaeqaaOGaeiykaKIaeiyxa0faaa@5873@

The resulting estimates can be treated as preliminary estimates and it is possible to return to point (2). The algorithm should be iterated at least once and for each individual i the final estimates are denoted with βiGLS
 MathType@MTEF@5@5@+=feaafiart1ev1aqatCvAUfKttLearuWrP9MDH5MBPbIqV92AaeXatLxBI9gBaebbnrfifHhDYfgasaacH8akY=wiFfYdH8Gipec8Eeeu0xXdbba9frFj0=OqFfea0dXdd9vqai=hGuQ8kuc9pgc9s8qqaq=dirpe0xb9q8qiLsFr0=vr0=vr0dc8meaabaqaciaacaGaaeqabaqabeGadaaakeaacqaHYoGydaWgaaWcbaGaeeyAaK2aaSbaaWqaaiabbEeahjabbYeamjabbofatbqabaaaleqaaaaa@336C@.

#### STAGE 2

In this stage it is assumed that the estimates βiGLS
 MathType@MTEF@5@5@+=feaafiart1ev1aqatCvAUfKttLearuWrP9MDH5MBPbIqV92AaeXatLxBI9gBaebbnrfifHhDYfgasaacH8akY=wiFfYdH8Gipec8Eeeu0xXdbba9frFj0=OqFfea0dXdd9vqai=hGuQ8kuc9pgc9s8qqaq=dirpe0xb9q8qiLsFr0=vr0=vr0dc8meaabaqaciaacaGaaeqabaqabeGadaaakeaacqaHYoGydaWgaaWcbaGaeeyAaK2aaSbaaWqaaiabbEeahjabbYeamjabbofatbqabaaaleqaaaaa@336C@ are treated as they were known. So the population estimates of the vector β and of the variance-covariance matrix D are given by the sample mean and the sample variance-covariance matrix:

β^=1m∑i=1mβ^iGLSandD^=1(m−1)∑i=1m(β^iGLS−β^)T(β^iGLS−β^).
 MathType@MTEF@5@5@+=feaafiart1ev1aaatCvAUfKttLearuWrP9MDH5MBPbIqV92AaeXatLxBI9gBaebbnrfifHhDYfgasaacH8akY=wiFfYdH8Gipec8Eeeu0xXdbba9frFj0=OqFfea0dXdd9vqai=hGuQ8kuc9pgc9s8qqaq=dirpe0xb9q8qiLsFr0=vr0=vr0dc8meaabaqaciaacaGaaeqabaqabeGadaaakeaafaqabeqadaaabaGafqOSdiMbaKaacqGH9aqpdaWcaaqaaiabigdaXaqaaiabb2gaTbaadaaeWbqaaiqbek7aIzaajaWaaSbaaSqaaiabbMgaPnaaBaaameaacqqGhbWrcqqGmbatcqqGtbWuaeqaaaWcbeaaaeaacqqGPbqAcqGH9aqpcqaIXaqmaeaacqqGTbqBa0GaeyyeIuoaaOqaaiabbggaHjabb6gaUjabbsgaKbqaaiqbbseaezaajaGaeyypa0ZaaSaaaeaacqaIXaqmaeaacqGGOaakcqqGTbqBcqGHsislcqaIXaqmcqGGPaqkaaWaaabCaeaacqGGOaakcuaHYoGygaqcamaaBaaaleaacqqGPbqAdaWgaaadbaGaee4raCKaeeitaWKaee4uamfabeaaaSqabaaabaGaeeyAaKMaeyypa0JaeGymaedabaGaeeyBa0ganiabggHiLdGccqGHsislcuaHYoGygaqcaiabcMcaPmaaCaaaleqabaGaeeivaqfaaOGaeiikaGIafqOSdiMbaKaadaWgaaWcbaGaeeyAaK2aaSbaaWqaaiabbEeahjabbYeamjabbofatbqabaaaleqaaOGaeyOeI0IafqOSdiMbaKaacqGGPaqkaaGaeiOla4caaa@6B26@

## Competing interests

The author(s) declare that they have no competing interests.

## Authors' contributions

SP: mathematical modelling, statistical analysis, drafting of the manuscript;

PP: mathematical modeling;

ADG: mathematical modeling, drafting of the manuscript;

All authors read and approved the final manuscript.
